# Green tea extract for treatment of cancers: A systematic review protocol

**DOI:** 10.1097/MD.0000000000015117

**Published:** 2019-04-12

**Authors:** Kai Cheng, Nan-Nan Chi, Jun-Dong Liu

**Affiliations:** a Second Ward of Gastroenterology Department; b Department of Pharmacy, First Affiliated Hospital of Jiamusi University, Jiamusi, China.

**Keywords:** cancer, efficacy, green tea extract, safety, systematic review

## Abstract

**Background:**

Previous clinical studies suggested that green tea extract (GTE) may benefit patients with a variety of cancers. However, its efficacy is still inconclusive. Thus, the objective of this study will systematically collate the clinical studies testing its efficacy and safety for cancers.

**Methods:**

We will perform a systematic review of clinical studies assessing the efficacy of GTE in variety of cancers. We will search Cochrane Central Register of Controlled Trials (CENTRAL), EMBASE, MEDILINE, Cumulative Index to Nursing and Allied Health Literature (CINAHL), Allied and Complementary Medicine Database (AMED), and Chinese Biomedical Literature Database (CBM) using a comprehensive strategy. We will also screen the reference lists of relevant studies to identify any additional studies for potential inclusion. All databases will be searched up to February 1, 2019. All eligible case-control studies and randomized controlled trials will be included in this study. Two independent authors will review all searched literature. Upon inclusion of trials, we will extract data by using a predefined standardized form. The risk of bias assessment will be evaluated by using Cochrane risk of bias tool. We will use RevMan 5.3 software to pool the data and carry out meta-analysis.

**Results:**

The primary outcome includes overall response rate. The secondary outcomes comprise of overall survival, progression-free survival, the disease control rate, and any adverse events.

**Conclusions:**

The results of this study will contribute to the understanding of the efficacy of GTE in the setting of cancers and promote future research of GTE in patients with cancers.

**Dissemination and ethics:**

The results of this systematic review are expected to be published through peer-reviewed journals. This study does not need ethic approval, because it does not utilize individual patient data.

**Systematic review registration:**

PROSPERO CRD42019125111.

## 1 Introduction

Cancers are lethal disorder with poor outcomes and increasing incidence around the world.^[1,2]^ There are more than 100 types of cancer, such as lung cancer, breast cancer, prostate cancer, lymphoma, gastric cancer, ovarian cancer, and so on.^[3]^ Symptoms often vary according to the different types of cancer. A variety of factors contribute to the cancers, including, alcohol, obesity, dietary factors, and infection.^[4,5]^ The cancer management may include chemotherapy, radiation, and/or surgery.^[6,7]^ However, all those treatments still have limited efficacy, and also accompany lots of toxicities.^[8]^ Therefore, more potential therapies are still urgently needed for patients with cancers.

Green tea is a very popular and widely consumed beverage globally.^[9,10]^ Previous studies have reported that highest green tea intake can significantly lower cancer risk.^[9–11]^ It contains several catechin components, also known as green tea extracts (GTE), including Epigallocatechin gallate, Epigallocatechin, Epicatechin-3-gallate, and Epicatechin.^[12]^ Several preclinical studies of green tea and its components are reported to show promising efficacy for the growth inhabitation of tumors with fewer adverse events.^[13,14]^ Furthermore, a numerous of clinical trials have reported that GTE can be used to treat a large range of cancers effectively, including breast cancer, colorectal cancer, prostate cancer, ovarian cancer, liver cancer, lung cancer, lymphoma, gastric cancer, and pancreatic cancer.^[9,10,15–29]^ Presently, no systematic review study has assessed the efficacy and safety of GTE for patients with a variety of cancers. Thus, this systematic review will first investigate the efficacy and safety of GTE for cancers.

## 2 Methods

### 2.1 Objective

This systematic review aims to assess the efficacy and safety of GTE for patients with cancers.

### 2.2 Study registration

The protocol of this study has been registered in PROSPERO with CRD42019125111.

### 2.3 Inclusion criteria for study selection

#### 2.3.1 Types of studies

All qualified randomized controlled trials (RCTs) and case-control studies will be included in this study without language or publication restrictions. However, non-clinical studies, case studies, uncontrolled studies will not be included.

#### 2.3.2 Types of patients

Patients who are clinically diagnosed with cancers, including breast cancer, colorectal cancer, prostate cancer, ovarian cancer, liver cancer, lung cancer, lymphoma, gastric cancer, and pancreatic cancer will all be fully considered for inclusion without the restrictions of race, gender, and age. However, patients with other severe disorders that may affect the outcome measurements will be excluded, such as acute heart failure, severe stroke and so on.

#### 2.3.3 Types of interventions

Any types of GTE therapy will be considered for inclusion, except the combination of GTE with other interventions. Control therapy can be any treatments, except the GTE, or any kinds of green tea.

#### 2.3.4 Types of outcome measurements

The primary outcome is measured by overall response rate (ORR). The secondary outcomes are assessed by overall survival (OS), progression-free survival (PFS), the disease control rate (DCR), and any adverse events.

### 2.4 Search methods for the identification of studies

#### 2.4.1 Search electronic bibliographic databases

We will search Cochrane Central Register of Controlled Trials (CENTRAL), EMBASE, MEDLINE, the Cumulative Index to Nursing and Allied Health Literature (CINAHL), the Allied and Complementary Medicine Database (AMED), and Chinese databases of Chinese Biomedical Literature Database (CBM) up to February 1, 2019 using a comprehensive strategy. Reference lists of relevant studies will also be identified for potential consideration. The sample of comprehensive search strategy for CENTRAL is presented in Table 1. Similar comprehensive search strategies will also applied to any other electronic databases.

**Table 1:**
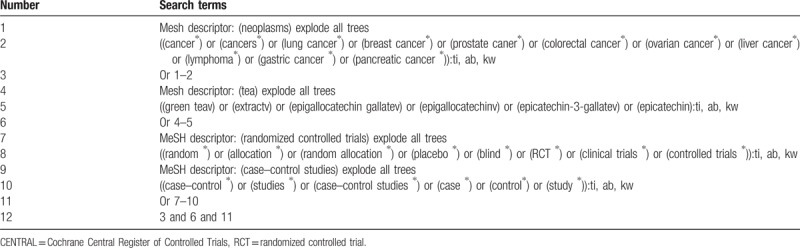
Search strategy for CENTRAL database.

#### 2.4.2 Search for other resources

References lists of relevant trials will also be searched to avoid missing any potential studies.

### 2.5 Data collection and extraction

#### 2.5.1 Study selection

**Figure 1:**
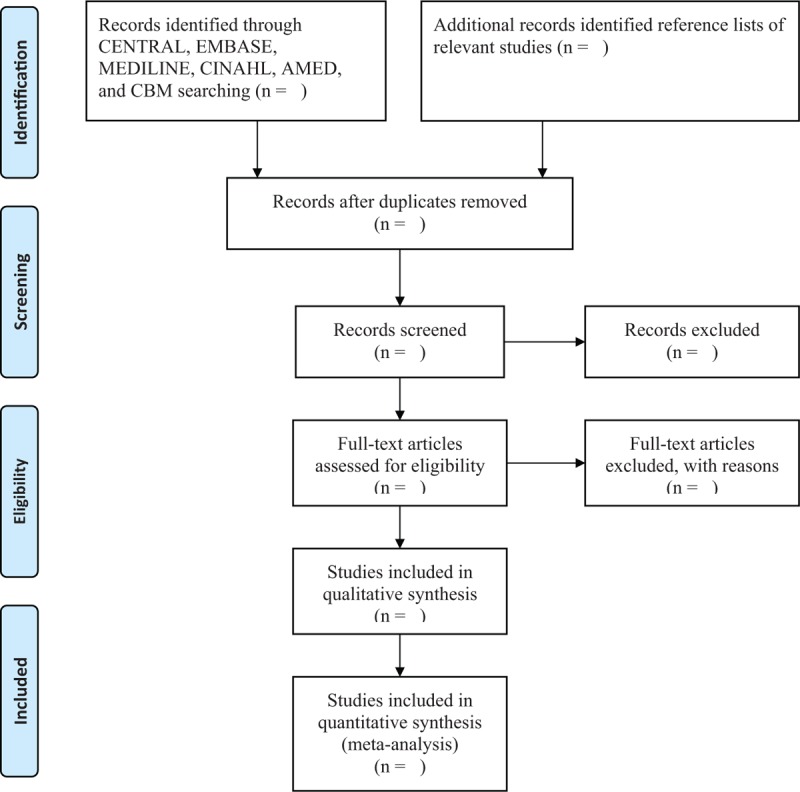
Flowchart of study selection.

Two authors will independently screen the title and abstract of all identified studies for eligibility. Then, full-texts will be read for further selection in accordance with the predefined eligibility criteria. Any disagreements between 2 authors will be solved by consulting a third author. The whole process of study selection will be presented in Figure 1.

#### 2.5.2 Data extraction and management

Two authors will independently extract data according to the predefined and standardized data extraction form. This form comprises of the data of study characteristics, such as title, first author, published year, location; patient populations, such as race, age, gender, eligibility criteria; study design, such as sample size, randomization, concealment, and blinding; interventions, such as dosage, frequency, and duration; and outcomes, such as primary, secondary, and safety outcome measurements. All divergences regarding the data extraction between 2 authors will be solved by a third author through discussion.

#### 2.5.3 Dealing with missing data

Any missing data will be inquired by contacting the primary authors. If we can not receive those data, then we will only analyze the available data and will discuss its potential impacts.

### 2.6 Risk of bias assessment

We will use Cochrane Risk of Bias Tool to evaluate the risk of bias for each included study. This tool assesses the risk of bias in 7 domains, and each domain will be determined as high, unclear, or low risk of bias. Any dissimilarity regarding the risk of bias assessment between 2 authors will be settled down by consensus with a third author.

### 2.7 Statistical analysis

RevMan 5.3 software will be utilized to pool the data and conduct meta-analysis in this study.

#### 2.7.1 Measurement of treatment effect

Dichotomous data will be shown as risk ratio and 95% confidence intervals (CIs). Continuous data will be demonstrated as mean difference or standardized mean difference and 95% CIs.

#### 2.7.2 Assessment of heterogeneity

Cochrane *I*^2^ test will be used to assess the heterogeneity. If the value of *I*^2^ >50%, significant heterogeneity is considered. Otherwise, if the value of *I*^2^ ≤50%, acceptable heterogeneity is considered.

#### 2.7.3 Data synthesis

If acceptable heterogeneity will be detected, a fixed-effect model will be used to pool and meta-analysis will be performed. Otherwise, if significant heterogeneity will be identified, a random-effect model will be applied, and subgroup analysis will be carried out. Meta-analysis will be conducted according to the results of subgroup analysis. If there is still significant heterogeneity after the subgroup analysis, meta-analysis will not be conducted. However, a narrative summary will be described.

#### 2.7.4 Subgroup analysis

Subgroup analysis will be carried out to identity any reasons that may cause the substantial heterogeneity. It will be performed based on the different treatments, controls, and outcome measurements.

#### 2.7.5 Sensitivity analysis

Sensitivity analysis will conducted to check the stability of the pooled results by removing the low quality of included studies.

#### 2.7.6 Publication biases

If there will be more than 10 qualified studies are included, Funnel plot and Egger linear regression test will be operated to identify any potential publication biases.

## 3 Discussion

Currently, no systematic review has been performed regarding the efficacy and safety of GTE for the treatment of patients with cancers. In this systematic review, we will first investigate the efficacy and safety of GTE for a variety of cancers. The results of this study will summarize the latest evidence for assessing the efficacy and safety of GTE for the treatment of cancers. Its findings may provide helpful evidence for both patients and clinicians.

## Author contributions

**Conceptualization:** Kai Cheng, Nan-Nan Chi.

**Data curation:** Kai Cheng, Nan-Nan Chi, Jun-Dong Liu.

**Formal analysis:** Nan-Nan Chi, Jun-Dong Liu.

**Funding acquisition:** Kai Cheng.

**Investigation:** Kai Cheng.

**Methodology:** Nan-Nan Chi, Jun-Dong Liu.

**Project administration:** Kai Cheng.

**Resources:** Nan-Nan Chi, Jun-Dong Liu.

**Software:** Nan-Nan Chi, Jun-Dong Liu.

**Supervision:** Kai Cheng.

**Validation:** Kai Cheng, Nan-Nan Chi.

**Visualization:** Kai Cheng.

**Writing – original draft:** Kai Cheng, Nan-Nan Chi, Jun-Dong Liu.

**Writing – review & editing:** Kai Cheng, Nan-Nan Chi, Jun-Dong Liu.

## References

[1] De Flora S La Maestra S Epidemiology of cancers of infectious origin and prevention strategies. J Prev Med Hyg 2015;56:E15–20. 26789827 PMC4718340

[2] Koubková L Hrstka R Dobes P Second primary cancers - causes, incidence and the future. Klin Onkol 2014;27:11–7. 24635432 10.14735/amko201411

[3] Denis H Davoine C Bermudez E Specific immunotherapies in the treatment of cancers. Bull Cancer 2019;106:37–47. 30638899 10.1016/j.bulcan.2018.12.007

[4] Yang L Drake BF Colditz GA Obesity and other cancers. J Clin Oncol 2016;34:4231–7. 27903157 10.1200/JCO.2016.68.4837

[5] Lee PN Thornton AJ Hamling JS Epidemiological evidence on environmental tobacco smoke and cancers other than lung or breast. Regul Toxicol Pharmacol 2016;80:134–63. 27321059 10.1016/j.yrtph.2016.06.012

[6] Mohiuddin M Regine WF Stevens J Combined intraoperative radiation and perioperative chemotherapy for unresectable cancers of the pancreas. J Clin Oncol 1995;13:2764–8. 7595736 10.1200/JCO.1995.13.11.2764

[7] Bernier J Poortmans PM Surgery and radiation therapy of triple-negative breast cancers: from biology to clinics. Breast 2016;28:148–55. 27318170 10.1016/j.breast.2016.05.014

[8] Cetina-Pérez L Serrano-Olvera A Flores-Cisneros L Epidemiological profile, gastrointestinal toxicity, and treatment of pelvic cancers in patients managed with radiotherapy to the abdominal pelvic area. Rev Invest Clin 2018;70:112–6. 29943771 10.24875/RIC.18002528

[9] Hu Y McIntosh GH Le Leu RK Supplementation with Brazil nuts and green tea extract regulates targeted biomarkers related to colorectal cancer risk in humans. Br J Nutr 2016;116:1901–11. 27923410 10.1017/S0007114516003937

[10] Samavat H Dostal AM Wang R The Minnesota Green Tea Trial (MGTT), a randomized controlled trial of the efficacy of green tea extract on biomarkers of breast cancer risk: study rationale, design, methods, and participant characteristics. Cancer Causes Control 2015;26:1405–19. 26206423 10.1007/s10552-015-0632-2 PMC4567901

[11] Imai K Suga K Nakachi K Cancer-preventive effects of drinking green tea among a Japanese population. Prev Med 1997;26:769–75. 9388788 10.1006/pmed.1997.0242

[12] Mattoli L Mercati V Burico M Experimental evidence of the presence of bimolecular caffeine/catechin complexes in green tea extracts. J Nat Prod 2018;81:2338–47. 30372064 10.1021/acs.jnatprod.8b00168

[13] Fujiki H Suganuma M Okabe S Japanese green tea as a cancer preventive in humans. Nutr Rev 1996;54:S67–70. 9110578 10.1111/j.1753-4887.1996.tb03821.x

[14] Yoshizawa S Horiuchi T Fujiki H Antitumor promoting activity of ())-epigallocatechin gallate, the main constituent of “tannin” in green tea. Phytother Res 1987;1:44–7.

[15] Samavat H Ursin G Emory TH A randomized controlled trial of green tea extract supplementation and mammographic density in postmenopausal women at increased risk of breast cancer. Cancer Prev Res (Phila) 2017;10:710–8. 28904061 10.1158/1940-6207.CAPR-17-0187 PMC7337967

[16] Lazzeroni M Guerrieri-Gonzaga A Gandini S A presurgical study of lecithin formulation of green tea extract in women with early breast cancer. Cancer Prev Res (Phila) 2017;10:363–70. 28400479 10.1158/1940-6207.CAPR-16-0298

[17] Dostal AM Samavat H Bedell S The safety of green tea extract supplementation in postmenopausal women at risk for breast cancer: results of the Minnesota Green Tea Trial. Food Chem Toxicol 2015;83:26–35. 26051348 10.1016/j.fct.2015.05.019 PMC4540665

[18] Kumar NB Pow-Sang J Egan KM Randomized, placebo-controlled trial of green tea catechins for prostate cancer prevention. Cancer Prev Res (Phila) 2015;8:879–87. 25873370 10.1158/1940-6207.CAPR-14-0324 PMC4596745

[19] Crew KD Ho KA Brown P Effects of a green tea extract, Polyphenon E, on systemic biomarkers of growth factor signalling in women with hormone receptor-negative breast cancer. J Hum Nutr Diet 2015;28:272–82. 24646362 10.1111/jhn.12229 PMC4205214

[20] Trudel D Labbé DP Araya-Farias M A two-stage, single-arm, phase II study of EGCG-enriched green tea drink as a maintenance therapy in women with advanced stage ovarian cancer. Gynecol Oncol 2013;131:357–61. 23988418 10.1016/j.ygyno.2013.08.019

[21] Stendell-Hollis NR Thomson CA Thompson PA Green tea improves metabolic biomarkers, not weight or body composition: a pilot study in overweight breast cancer survivors. J Hum Nutr Diet 2010;23:590–600. 20807303 10.1111/j.1365-277X.2010.01078.x PMC2966548

[22] Wang P Aronson WJ Huang M Green tea polyphenols and metabolites in prostatectomy tissue: implications for cancer prevention. Cancer Prev Res (Phila) 2010;3:985–93. 20628004 10.1158/1940-6207.CAPR-09-0210 PMC3163459

[23] Brausi M Rizzi F Bettuzzi S Chemoprevention of human prostate cancer by green tea catechins: two years later. A follow-up update. Eur Urol 2008;54:472–3. 18406041 10.1016/j.eururo.2008.03.100

[24] Bettuzzi S Brausi M Rizzi F Chemoprevention of human prostate cancer by oral administration of green tea catechins in volunteers with high-grade prostate intraepithelial neoplasia: a preliminary report from a one-year proof-of-principle study. Cancer Res 2006;66:1234–40. 16424063 10.1158/0008-5472.CAN-05-1145

[25] Luo H Tang L Tang M Phase IIa chemoprevention trial of green tea polyphenols in high-risk individuals of liver cancer: modulation of urinary excretion of green tea polyphenols and 8-hydroxydeoxyguanosine. Carcinogenesis 2006;27:262–8. 15930028 10.1093/carcin/bgi147

[26] Choan E Segal R Jonker D A prospective clinical trial of green tea for hormone refractory prostate cancer: an evaluation of the complementary/alternative therapy approach. Urol Oncol 2005;23:108–13. 15869995 10.1016/j.urolonc.2004.10.008

[27] Laurie SA Miller VA Grant SC Phase I study of green tea extract in patients with advanced lung cancer. Cancer Chemother Pharmacol 2005;55:33–8. 15309507 10.1007/s00280-004-0859-1

[28] The Indian-US Head and Neck Cancer Cooperative Group. Green tea and leukoplakia. Am J Surg 1997;174:552–5. 9374236

[29] Dai Q Ji BT Jin F Case-control study of pancreatic cancer in Shanghai population—relationship between pancreatic cancer and tobacco, alcohol and green tea. Tumor 1996;1:5–10.

